# Complete genome sequence of *Mycobacterium tuberculosis* variant *bovis* strain ushi10, a tuberculin production strain used in Japan

**DOI:** 10.1128/mra.00749-26

**Published:** 2026-10-15

**Authors:** Koume Mastubara, Takashi Mada, Kazuhiro Sata, Kazumasa Shiraiwa, Satoko Kawaji, Daisuke Takamatsu, Reiko Nagata

**Affiliations:** 1 National Institute of Animal Health, National Agriculture and Food Research Organization 13516, Tsukuba, Ibaraki, Japan; University of Maryland School of Medicine, Baltimore, Maryland, USA

**Keywords:** *Mycobacterium tuberculosis*, tuberculin, production strain, Japan, complete genome

## Abstract

Here, we report the complete genome sequence of *Mycobacterium tuberculosis* variant *bovis* strain ushi10, a production strain used to manufacture tuberculin for bovine tuberculosis diagnosis in Japan. This genomic resource supports the consistent production and standardization of tuberculin.

## ANNOUNCEMENT

Since 1901, a continuous surveillance program using the tuberculin skin test has led to the eradication of bovine tuberculosis (bTb) from cattle in Japan (1). The tuberculin antigen is prepared from designated production strains of *Mycobacterium tuberculosis* complex (2). In Japan, the ushi10 strain has historically been used for this purpose.

In this study, we report the complete genome sequence of ushi10. ushi10 was isolated from the mediastinal lymph nodes of a bTb-affected cow at the National Veterinary Assay Laboratory (currently the National Institute of Animal Health, National Agriculture and Food Research Organization, Japan) in 1933 and designated as a production strain in April 1950. The lyophilized batch of ushi10 used here was produced in May 1970. The strain was cultured on Ogawa medium at 37°C for 4 months (3).

Genomic DNA was extracted from a subculture on Ogawa medium by suspending cells in distilled water and heat-inactivating them at 80°C for 20 min. DNA was then purified using the Johne-Pure-Spin kit (FASMAC, Japan) as described by Ueno et al. (4). DNA quality (DIN, 6.9) and concentration were assessed by agarose gel electrophoresis and Qubit, respectively.

Short-read sequencing was performed using an Illumina NovaSeq X system (Illumina, USA) with a 151-bp paired-end read setting. A TruSeq DNA PCR-Free library (approximately 350-bp inserts; Illumina) was prepared. Paired short-read sequencing yielded 30,470,230 + 30,470,230 reads for a total of 4,601,004,730 + 4,601,004,730 bp. Quality checks of these reads were performed using Sequali v1.0.2 (5), and adapter sequences and low-quality reads (<Q20) were removed using Atria v4.1.4 (6).

Long-read sequencing was performed on the Oxford Nanopore MinION Mk1D system with an R10.4.1 flow cell, using a rapid barcoding kit (SQK-RBK114.24) (Oxford Nanopore Technologies, UK) for library preparation. All subsequent data analyses were performed using default parameters except where otherwise noted. Basecalling was performed using Dorado v1.0.2 (7) with the super-accurate (SUP) model (dna_r10.4.1_e8.2_400bps_sup@v5.2.0). Long-read sequencing yielded 667,285 reads totaling 1,528,612,434 bp, with a read N_50_ value of 4,693 bp. Long reads were trimmed using NanoFilt v2.8.0 (8) and filtered via Filtlong v0.2.1 (9) (--keep_percent 90, --target_bases 500000000). Trimmed long reads were assembled *de novo* using Flye v2.9.6 (10), Miniasm v0.3-r179 combined with Minipolish v0.2.0 (11), and Raven v1.8.3 (12). A consensus assembly was generated using Trycycler v0.5.5 (13) and polished with long reads using Dorado polish implemented in Dorado v1.0.2. Remaining errors were corrected with short reads using NextPolish v1.4.1 (14) and Polypolish v0.6.0 (15, 16).

The complete ushi10 genome consists of a single circular chromosome (4,340,782 bp, 65.64% GC content), with 203× (long reads) and 1,911× (short reads) coverage. Trycycler v0.5.5 (13) was used to confirm chromosome circularity and rotate it to start at *dnaA*. Using DFAST-core v1.3.7 for genome annotation (17), we identified a total of 3,992 protein-coding genes, 3 rRNA genes, and 52 tRNA genes. CheckM v1.2.5 estimated 99.78% completeness and 0.00% contamination (18). Average nucleotide identity and digital DNA-DNA hybridization values versus the *M. tuberculosis* variant *bovis* strain AN5 (GCF_000488935.1) were 99.79% and 99.30%, respectively (19, 20), confirming its taxonomic assignment.

## Data Availability

The raw sequence-read data of ushi10 from MinION and NovaSeq X are deposited in the DDBJ/GenBank/EMBL database under BioProject accession number PRJDB40385, BioSample accession number SAMD01819996, and DRR accession numbers DRR909656 and DRR909657. The complete genome sequence and DFAST-core-based annotation are deposited with the accession number AP046124.
